# Revealing the functionality of hypothetical protein KPN00728 from *Klebsiella pneumonia*e MGH78578: molecular dynamics simulation approaches

**DOI:** 10.1186/1471-2105-12-S13-S11

**Published:** 2011-11-30

**Authors:** Sy Bing Choi, Yahaya M Normi, Habibah A Wahab

**Affiliations:** 1 Pharmaceutical Design and Simulation (PhDS) Laboratory, School of Pharmaceutical Sciences, Universiti Sains Malaysia, 11800 Minden, Pulau Pinang, Malaysia; 2 Department of Cell and Molecular Biology, Faculty of Biotechnology and Biomolecular Sciences, Universiti Putra Malaysia, 43400 Serdang, Selangor, Malaysia

## Abstract

**Background:**

Previously, the hypothetical protein, KPN00728 from *Klebsiella pneumoniae* MGH78578 was the Succinate dehydrogenase (SDH) chain C subunit via structural prediction and molecular docking simulation studies. However, due to limitation in docking simulation, an in-depth understanding of how SDH interaction occurs across the transmembrane of mitochondria could not be provided.

**Results:**

In this present study, molecular dynamics (MD) simulation of KPN00728 and SDH chain D in a membrane was performed in order to gain a deeper insight into its molecular role as SDH. Structural stability was successfully obtained in the calculation for area per lipid, tail order parameter, thickness of lipid and secondary structural properties. Interestingly, water molecules were found to be highly possible in mediating the interaction between Ubiquinone (UQ) and SDH chain C via interaction with Ser27 and Arg31 residues as compared with earlier docking study. Polar residues such as Asp95 and Glu101 (KPN00728), Asp15 and Glu78 (SDH chain D) might have contributed in the creation of a polar environment which is essential for electron transport chain in Krebs cycle.

**Conclusions:**

As a conclusion, a part from the structural stability comparability, the dynamic of the interacting residues and hydrogen bonding analysis had further proved that the interaction of KPN00728 as SDH is preserved and well agreed with our postulation earlier.

## Background

In the genome map of an organism, there are genes which code for hypothetical proteins. They contribute about 20 to 40% of total proteins [1]. The only information can be obtained on hypothetical protein is from their nucleotide and amino acid sequences as rather few experimental data is found for this category of proteins. Despite many years of investigation, the annotations of these proteins have yet to progress significantly. Hence, these hypothetical proteins provide large research opportunities to scientists to elucidate their structures and functions especially those from pathogens [2].

Approximately 20% of 4776 protein coding genes of *Klebsiella pneumoniae* MGH78578 pathogen are classified as hypothetical proteins [3]. *K. pneumoniae* is an opportunistic pathogen which affects patients with weakened immune system and/or underlying diseases [4]. Elucidating the structures and functions of these hypothetical proteins will help to give insight to the possible roles and mechanisms of these proteins in relation to the pathogenesis or survivability of the pathogen. In addition to this, new functions may also emerge from protein complexes. All the information obtained can be a stimulant for further drug discovery efforts in the future.

Previously, via homology modeling and docking studies, we postulated that hypothetical protein KPN00728 (gi: 152969292) is the chain C subunit of Succinate dehydrogenase (SDH) [5]. In both eukaryotic and prokaryotic organisms, SDH plays an important role in the aerobic respiratory chain specifically in the Krebs cycle which occurs in the transmembrane (TM) region of mitochondria. Our previous study showed that KPN00728 has a missing region containing conserved amino acid residues important for Ubiquinone (UQ), the natural ligand of SDH and heme group binding. Secondary structure and TM topology analyses showed that KPN00728 adopts SDH (subunit C)-like structure. Evolutionary relationship across 7 other Enterobacteriaceae was analyzed and showed that they are highly conserved. Molecular docking simulation on the other hand showed that UQ docked well onto the built model (consisting of KPN00728 and the annotated SDH chain D-KPN00729). Formation of hydrogen bonds between UQ and Ser27, Arg31 (from KPN00728) and Tyr83 (from KPN00729) further reinforced that KPN00728 hypothetical protein together with KPN00729 preserved the functionality of UQ binding. This observation strongly supported the possibility that KPN00728 is indeed chain C of SDH.

Although docking simulations enabled us to understand the preferred orientation of UQ when bound to the built model to form a stable complex, there were however, limitations. In docking simulation, rigidity of the built protein model and target of docking location are defined by the user. Hence this decreases the degree of freedom of both interacting components during the simulation. Furthermore, results from docking can only provide a single snapshot of the ligand orientation which does not represent a global, real-time picture of the dynamics of the interactions. Therefore, in this present study, molecular dynamics simulation was employed to obtain an in-depth understanding of the structure and function of KPN00728 as chain C of SDH across a successfully built model of the membrane environment of mitochondria.

## Results and discussion

### Membrane structure and selection on type of membrane

**Figure 1:**
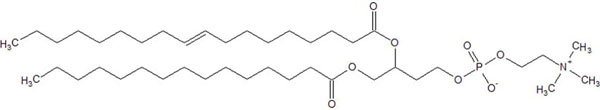
**Schematic representation of a single molecule of POPC 2D structure.** POPC consist of 2 main groups, a diacylglycerol and a phospholipids heap groups.

Membrane of different cells has a variety of composition [6]. In our previous study [5], we had proposed that KPN00728 is possibly the chain C of SDH [5]. SDH is a very important enzyme in the Krebs cycle. It is located at the inner mitochondrial membrane which is known to consist of Palmitoyloleoyl phosphatidylcholine (POPC), palmitoyl oleoyl phosphatidyl ethanolamine (POPE), palmitoyl oleoyl glycerophosphoserine (POPS), cholesterol and other trace compounds. Among lipids, POPC has the highest distribution of about 44% [6] and this formed the basis in selecting POPC as our model membrane (Figure 1).

### Stability of the system

**Figure 2:**
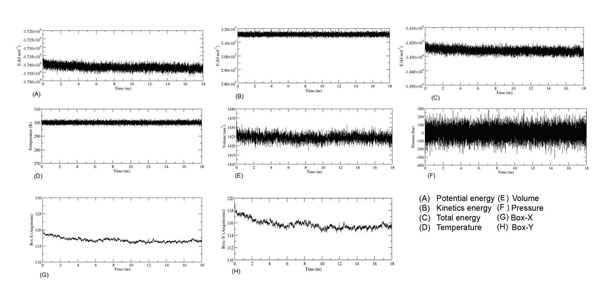
**Stability of the simulation system.** Stability was evaluated in term of the energies, temperature, volume, pressure and also box-x and box-y dimension of the simulation box as a function of simulation time.

The potential, kinetic and total energy profiles of the system are shown in Figure 2. The potential energy of the system decreased slowly at the first 2 ns of the production run but remained constant throughout the 18 ns thereafter. Similar profile was observed in the kinetic energy and total energy plots of the system. The temperature, pressure and volume profiles also indicated that the system had reached equilibration (Figure 2).

### Dynamic behaviour of Lipid membrane

The dynamics of the lipid membrane in this simulation was investigated to ascertain that our model is fully hydrated and comparable to experimental results as well as to previous simulations [7-9]. The properties investigated include lipid hydration, area per lipid, thickness of the membrane and order parameter of the hydrocarbon chains. It is clear from the results described below that our membrane adopted fully hydrated bilayer membrane behaviour.

**Figure 3:**
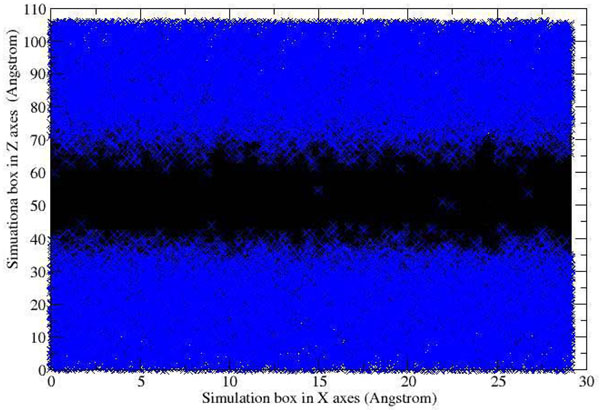
**Water distribution plot.** No significant amount of water molecules appeared in the hydrophobic lipid tail region.

**Figure 4:**
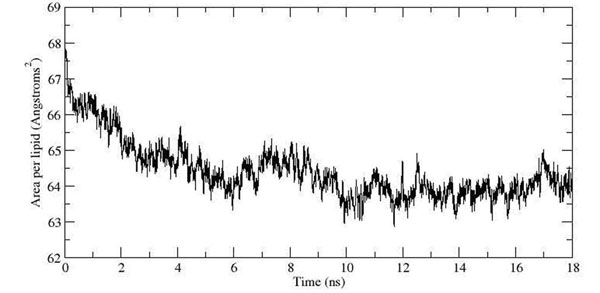
**Area per lipid plot**. Stability of the area per lipid was observed after 10 ns of the production simulation. It maintained at the average of 64.4Å.

**Figure 5:**
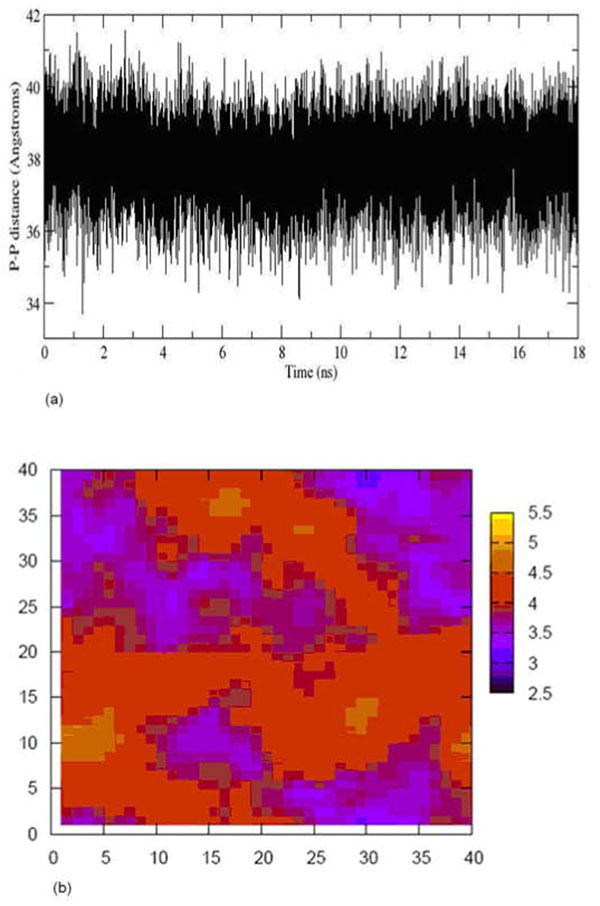
**Thickness evolution and distribution of POPC bilayer.** (a) Thickness of POPC bilayer versus time evolution. No significant fluctuation was observed throughout the entire 18 ns MD production run. (b) Thickness distribution of the POPC membrane layer of the simulation system.

In comparison with previous studies [10-12] our results showed that 69 molecules of water per lipid provide a fully hydrated membrane system (Table 1). The excessive waters molecules were added to ensure a fully hydrated membrane system and the water distribution across the simulation box in average structure had also indicated no significant water molecules present in the hydrophobic lipid tail region (Figure 3). The average area per lipid for the 18 ns production is 64 Å^2^ (Figure 4) which is close to the accepted experimental value and comparable to other simulations (62-64 Å^2^) [7-9,13-15] (Table 2). The thickness (P-P distance) of a typical membrane bilayer is about 40-50 Å [16,17]. No significant fluctuation of thickness of POPC membrane was observed during simulation (Figure 5b) and the average thickness of POPC calculated over the trajectory is ~38 Å (Figure 5a), in good agreement with the experimental-determined thickness (~37 Å) [18]. The average structure of 18 ns simulation was used to measure the distribution of the membrane thickness within the simulation box (Figure 5b). Therefore, by observing the average distribution of the membrane thickness along (Figure 5b) with the P-P distance of the membrane throughout the total simulation time, the thickness of the membrane is well correlated with the experimental value.

**Table 1:** Comparison of various POPC membrane protein systems with different hydration level

References	Number of lipid	Number of water	Water/lipid
Alamethicin helices in a bilayer and in solution: molecular dynamics simulations [10]	128	3467	27
Molecular dynamics study of the internal water molecules in vasopressin and oxytocin receptors [11]	120	3500	29
Combined monte carlo and molecular dynamics simulation of fully hydrated dioleyl and palmitoyl-oleyl phosphatidylcholine lipid bilayers [12]	128	4628	36

**Table 2:** Comparison of area per lipid in simulation and experimental value in previous studies

References	Area per lipid (Å^2^)		References
		
	Simulation	Experimental	
Lipid Models for United-Atom Molecular Dynamics Simulations of Proteins [8]	65.4±0.8	68.3	Structure of fully hydrated fluid phase lipid bilayers with monounsaturated chains [7]
Molecular characterization of gel and liquid-crystalline structures of fully hydrated POPC and POPE bilayers. [9]	~60.0-76.0	63.0 (310K)	Phosphatidylcholine acyl unsaturation modulates the decrease in interfacial elasticity induced by cholesterol [14]
Performance of the general amber force field in modeling aqueous POPC membrane bilayers [13]	50.0±0.43 54.8±0.25	62.0 (323K)	Structural information from multilamellar liposomes at full hydration: full q-range fitting with high quality X-ray data. [15]

The state of hydration is also related to the disorder of the membrane. Deuterium tail order parameter, S_cd_ can be calculated using the equation below:-

Where θ is the angle between the CD bond and the bilayer normal (bilayer molecular axis), and the angle brackets indicate that values are averaged over all equivalent atoms and over time.

**Figure 6:**
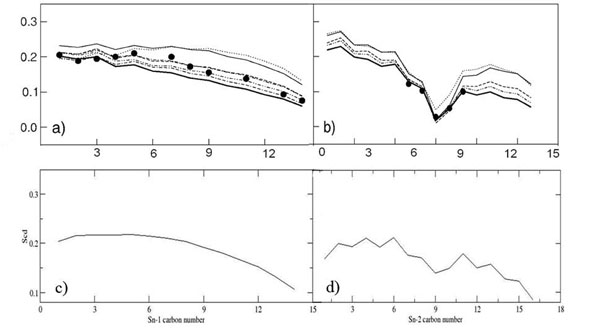
**Comparison of Deuterium tail order parameters for sn-1 and sn-2 POPC tail**. (a) and (b) are adapted from Sukit Leekumjorn; Amadeu K. Sum; *J. Phys. Chem. B* 2007, 111, 6026-6033. Similar profile is observed in our simulation system for both sn-1 and sn-2 POPC tail in (c) and (d).

Experimental data revealed that hydrated membrane bilayer has a |S_cd_| maximum value of 0.2 on *sn-1* tails (Figures 6a and b). Figure 6(c) and (d) shows the disorder of the alkyl chain of POPC, which clearly behaved similar to experimental results [9], where the carbon chain near the head group are more ordered and oriented compared to the terminals of POPC tails.

### Dynamics of succinate dehydrogenase

**Figure 7:**
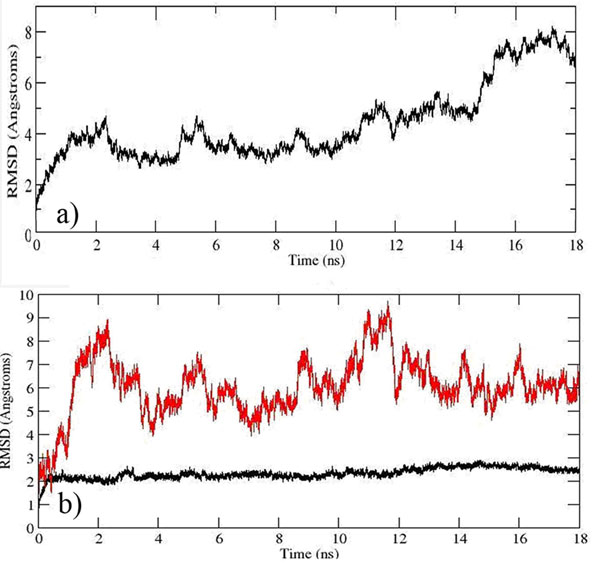
**(a) Overall backbone RMSD of the complete built model. (b)RMSD of the built model at TM region.** The RMSD in the TM region rises from 0 to ~2 Å at the first 0.3 ns of the simulation and remains stable around 2 Å after that. The cytoplasm region, on the other hand, was observed to shift significantly from 0 to ~9 Å during the first 2 ns of the simulation and fluctuates dramatically after 2ns until reach a relative equilibrium after 14 ns.

Structural stability of the built model was investigated based on properties such as root mean square deviation (RMSD), root mean square fluctuation (RMSF), radius of gyration and secondary structure of the model. RMSD of the built model measures the overall drift from its initial conformation during the simulation (Figure 7a). The RMSD of the backbone increased from ~1 Å at 0 ns to 4 Å at 1 ns but remained stable at 3-4 Å after 2 ns. However, significant deviation ranging from 4 to 8 Å occurred after 10 ns and this was probably due to the flexibility of the non-tranmembrane (non-TM) region of the model. To investigate this matter further, the built model was divided into TM and cytoplasmic regions. The cytoplasmic region consists of both loops and turns which extend out from the membrane into the cytoplasm. These loops and turns are more flexible, thus resulted in a higher RMSD value when compared to the helical bundle in the TM region (Figure 7b).

**Figure 8:**
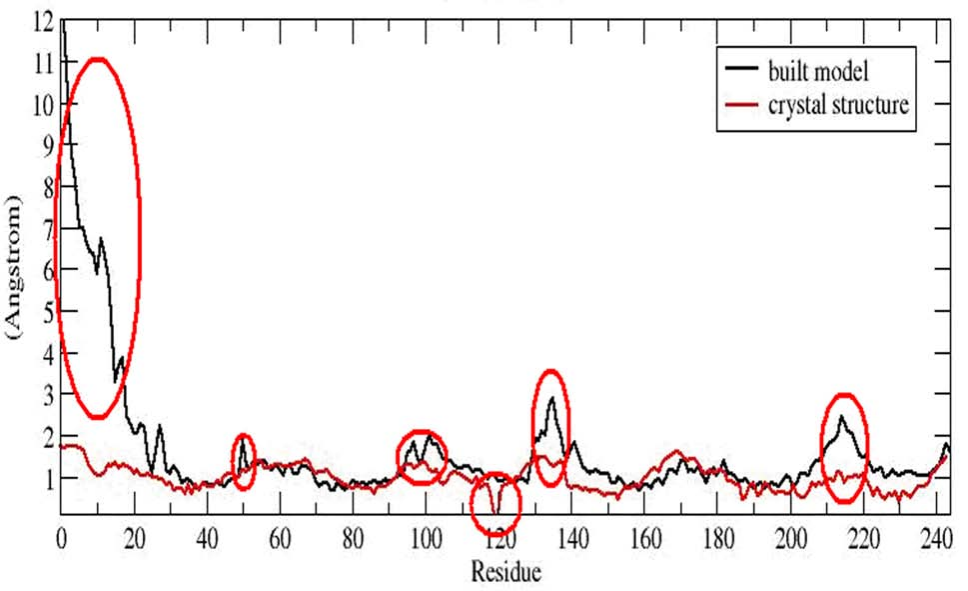
**Comparison of RMSF with crystal template with built model**. Red circle indicated high fluctuation. Higher fluctuation was seen in Ser47 as compared to the crystal structure (template) due to the fact that Ser47 extruded out from the lipid bilayer and exposed to the water. The coil regions such as residues 97-102, 129-138, 212-215 and the C terminal of chain D in the build model had showed also the high fluctuation as compare the helices in the structure. There are two missing residues at residues 119-120 in the crystal structure which cause the sudden decrease of RMSF occurred.

The flexibility of each residue can be inferred from RMSF for each residue. Despite the similarity of the simulated RMSF profile to that of the crystal structure of SDH chain C from *Escherichia coli* (Figure 8), high fluctuation occurred at the first 22 residues of KPN00728 (putative chain C) as they are located in the cytoplasm which allows this region to fluctuate robustly compared to the restrictive TM region. The fluctuation of the residues in the TM region decreased spontaneously as it adopted a more rigid structure in the TM region during the simulation.

**Figure 9:**
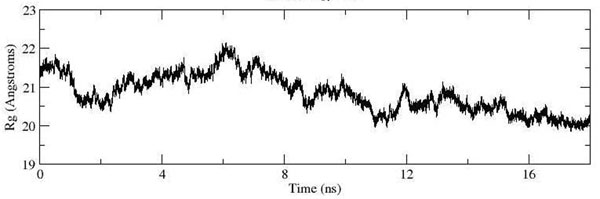
**Radius of gyration of the backbone built model against the time evolution.** SDH is considerably stable and no structural unfolding is observed. No significant changes are observed in term of its compactness.

**Figure 10:**
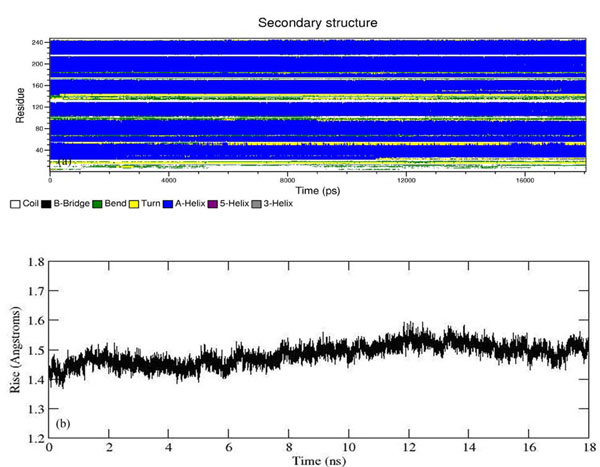
**(a) Time evolution of secondary structure throughout 18ns simulation. (b) Rise per residue of the model as a function of simulation time.** No significant fluctuation was observed in term of Rise per residue of the model indicated that the psi and phi angle remained stable during the simulation.

The stability of SDH can also be inferred from the calculated radius of gyration. No significant drift was observed and the gyration of the built model had fluctuated ~20 Å (Figure 9). This indicated that SDH was considerably stable and no structural unfolding was observed. This is also supported by the fact that the secondary structure element did not fluctuate significantly during the entire simulation time with the only major difference being observed at the beginning of both the postulated chain C helix bundle (Residues 1-20) and also at the beginning of chain D of SDH (residues 130-140) (Figure 10a). These residues apparently adopted random coil conformation. The fact that there was no collapse in the secondary structure of the built model indicated that the model was structurally stable throughout the entire simulation time. It should be noted that the calculated average helix radius was 3.4 ± 0.1 Å which was higher than the experimental helix radius value (~2.4 Å) [19] but the rise per residue in the helix (Figure 10b) was 1.5 Å which was comparable to experimental data [19-21]. The good agreement between our simulation and experimental data implied that our model and the simulation condition were acceptable and appropriate for further investigating the properties of hypothetical protein KPN00728 as chain C of SDH.

### Ubiquinone-SDH interaction

**Figure 11:**
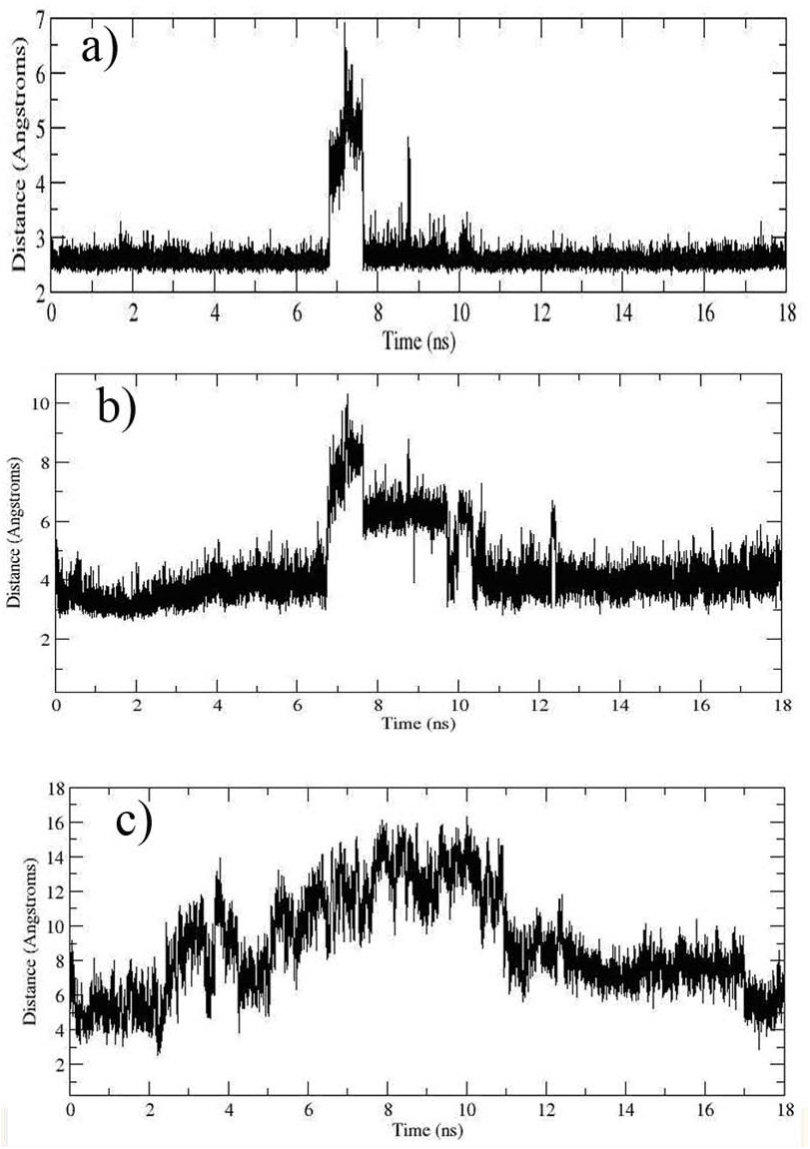
**Distance between the potential interacting residues with UQ**. (a) Distance between Tyr@OH and UQ@O4. (b) Distance between Arg31@NH1 and UQ@O2. (c) Distance between Ser27@OG and UQ@O3.

The formation of Hydrogen bonds (H-bond) between UQ and Tyr83@OH (SDH chain D), Ser27@OG (postulated chain C from KPN00728) and Arg31@NH1 (postulated chain C from KPN00728) is an important aspect for KPN00728 to preserve its functionality in UQ binding [5]. Table 3 shows the average distance between UQ and the binding site. Comparison between MD and docking results was done. The distance between Tyr83@OH and UQ@O1 was preserved at an average distance of 2.68 ± 0.49 Å (Figure 11a), indicating a high possibility H-bond formation between them. However UQ drifted further away from both Arg and Ser during the simulation (Figure 11b and c) to distances of 4.40 ± 1.26 Å and 8.83 ± 2.84 Å, respectively, thus decreasing the possibility for H-bond formation between them. As opposed to those observed in docking simulation, there were large shift of the distances between these two interacting residues and UQ at around 6-10 ns of the simulation (Figure 11). Further examination showed that the UQ binding site was located at the entrance of the two chains (chain C and chain D of SDH) in the TM area.

**Table 3:** Distance between Ubiquinone and Heme interacting

Interaction	MD result (Å)	Docking result (Å)
TYR83@OH and UQ@O1	2.68 ± 0.49	2.58
ARG31@NH1 and UQ@O2	4.40 ± 1.26	3.83
SER27@OG and UQ@O3	8.83 ± 2.84	2.68
HIS84@ND and HEME@FE	7.07 ± 0.43	3.25
HIS71@ND and HEME@FE	6.43 ± 0.79	1.29

**Figure 12:**
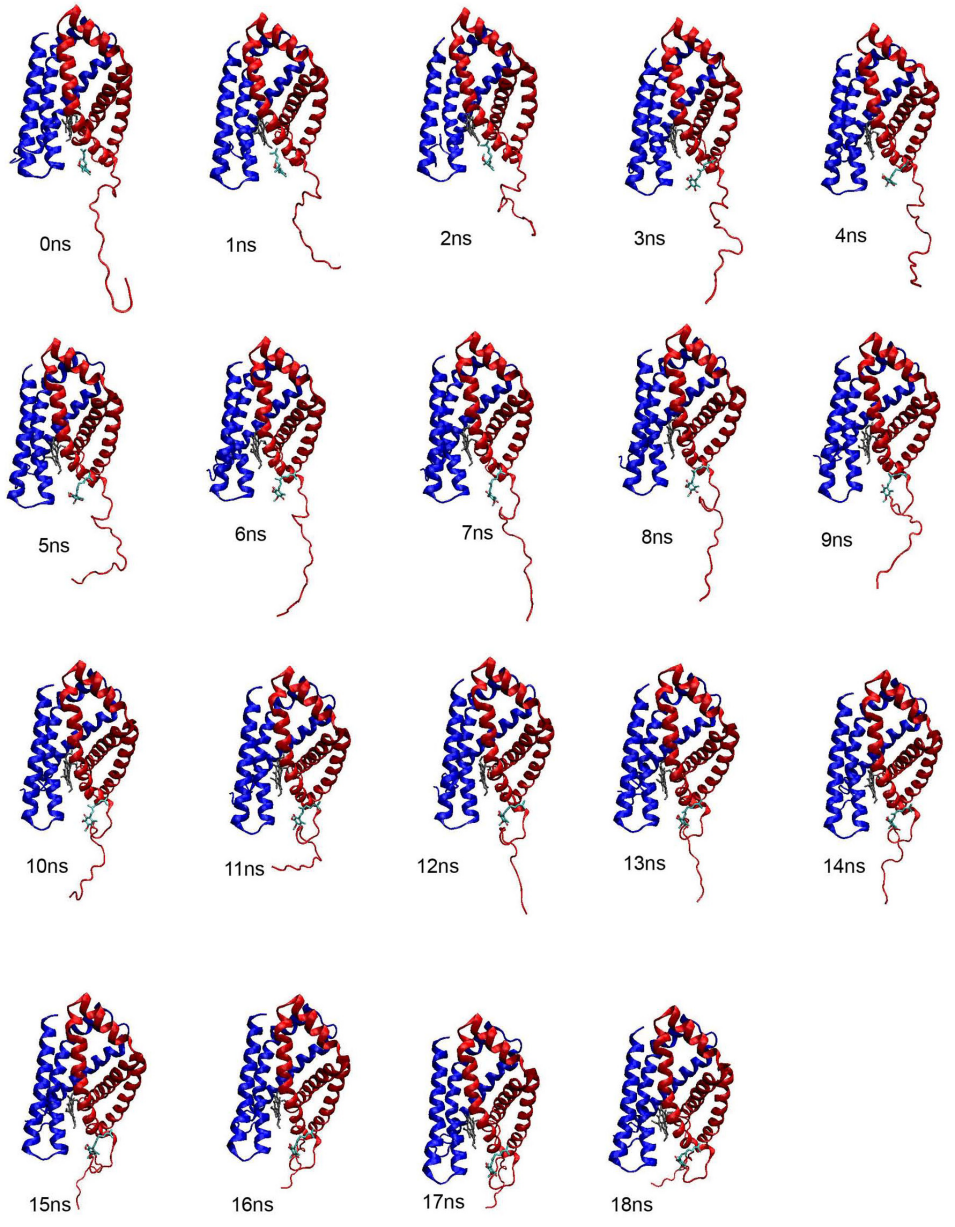
**Single snapshot of the built model sampled every one nanosecond of MD simulation time.** High flexibility of the cytoplasm region of the postulated SDH model was observed among these single snapshots. The cytoplasm region had started to fold from 10 ns onward and this might possible be the reason that UQ had moved in to the transmembrane region. KPN00728 is representing in red and chain D is indicated in blue.

**Figure 13:**
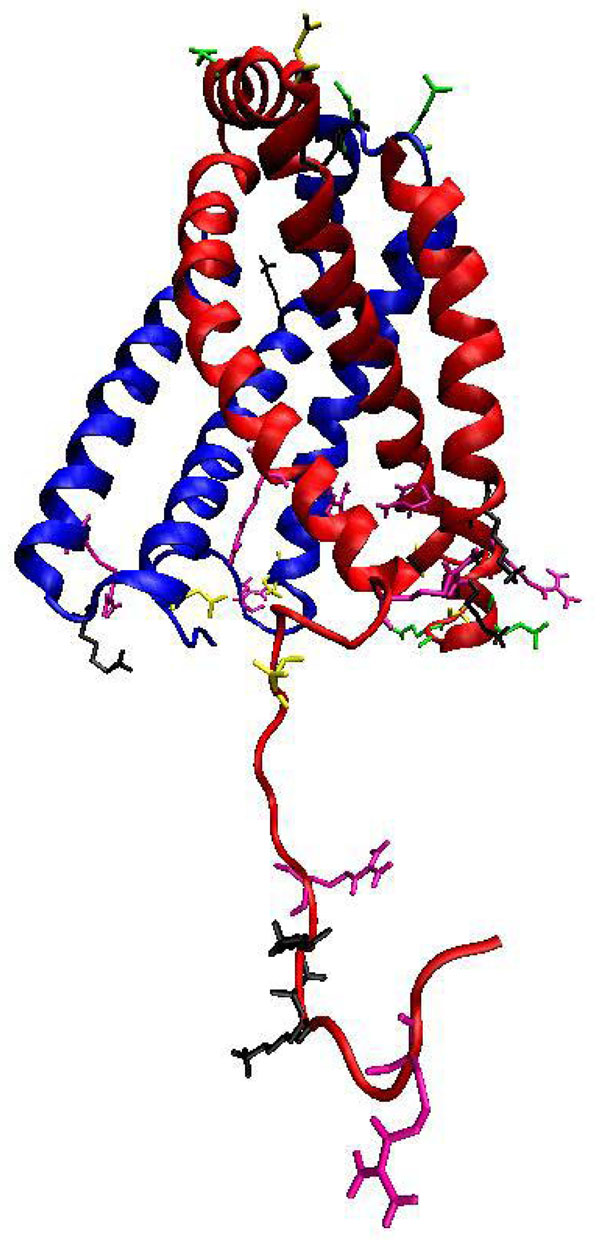
**Amino acids that contribute to the repulsive force between the loop and the entrance of the water channel.** Amino acids such as Asp (Yellow), Arg (Magenta), Glu (Green) and Lys (Gray) might be contributing towards the repulsion of the loop which found with high flexibility.

Residues 1 to 22 of the built model formed turn, loop and bend at the entrance of UQ binding site. Based on RMSF of the model (Figure 8), the cytoplasm region exhibited very high flexibility in terms of its conformation. During the simulation, the cytoplasmic region of the model appeared to move further away from the entrance of the UQ binding site from 6 ns onward and UQ also simultaneously had drifted further out from the binding site. However, ~7 ns onward, the cytoplasmic region had moved closer towards the entrance of the binding site. This might be due to the repulsive force exerted by the cytoplasmic loop (Figure 12). This repulsive force was postulated to be contributed by several polar residues which located at the entrance such as Lys, Arg and Asp. These residues are known to exert high repulsive force [22] (Figure 13). The cytoplasmic region may act as a door which guarded the movement of UQ from going in and out from the TM to the cytoplasm of *K. pneumoniae.*

### Solvation effect on the UQ binding

It was found that water molecules were present at the entrance and void area between KPN00728 and chain D of SDH (i.e. at binding site of UQ). The existence of water molecules at the void area between the protein and UQ promoted us to postulate that the binding of the UQ to protein may occur with the assistance of water molecules. Water might be responsible in causing the drift in the distance between them. The contribution of water molecules in the binding process was studied using radial distribution function (RDF) of all hydrogen bond acceptors in UQ, (O1, O2, O3 and O4) to the water molecule. In addition, RDF was also calculated on the interacting residues i.e. Ser27@OG, Arg31@NH1, Arg31@NH2 and Tyr83@OH (Table 4). UQ@O1 with OW had low RDF intensity of 0.07 at 3 Å as O1 formed a strong H-bond with Tyr83@OH throughout 18 ns simulation time. UQ@O4 and OW, on the other hand, showed the highest intensity among all other oxygen atoms and they acted as H-bond acceptor from UQ.

**Table 4:** Radial distribution function was done between the ubiquinone and protein interacting residues

Potential interacting residues	Intensity g (r)	Distance, r (Å)	Average number of particles
**Hydrogen acceptor at ubiquinone**			
UQ@O1-SOL@OW	0.07	3.00	0.054
UQ@O2-SOL@OW	0.06	3.02	0.058
UQ@O3-SOL@OW	0.28	3.52	0.101
UQ@O4-SOL@OW	1.09	3.39	0.090
**Interacting residues at built model**			
SER27@OG-SOL@OW	3.55	1.66	2.700
ARG31@NH1-SOL@OW	0.19	3.20	0.120
ARG31@NH2-SOL@OW	0.14	2.15	0.020
TYR83@OH-SOL@OW	0.07	3.40	0.075

RDF of Ser27@OG with OW also showed the highest intensity of 3.55 at 1.66 Å with an average number of water particles of ~2.77. This result also indicated that Ser27@OG might be able to form hydrogen bond with water molecules as the distance is within the H-bond cut-off value. Although Arg31@NH1 and Arg31@NH2 were also postulated to act as H-bond donors during interaction with UQ, the distance between Arg31@NH1 and UQ@O2 is far apart and the possibility of a H-bond formed between them is low. Thus, we suspect that water might play a role between them by forming water-mediating H-bond.

In RDF calculation, the intensity for both NH1 and NH2 groups from Arg31 with OW were low. The possibility of finding water molecules at 3.2 Å and 2.15 Å were as low as 0.19 and 0.14, respectively. Based on these results, the possibility of water to appear around both NH1 and NH2 from Arg31 is low. To further prove water molecules might be responsible in creating the drift of UQ from the interacting residues, which eventually eliminate the hydrogen bonding between UQ and the binding site residues, H-bond analysis between the interacting residues and UQ with water was performed.

A 5 Å shell was set around the binding site residues from SDH and UQ (Table 5). The analyses showed a minimum of one water mediated H-bond was found in more than 90.0% of the simulation time around Ser27@OG and UQ@O3. In addition, 39.4% of the trajectory appeared to have two water-mediating H-bonds. For UQ@O3, more than 55.0% of the trajectory consist at least two water-mediating H-bonds between UQ@O3 and protein. Only 0.74% and 0.68% of the trajectories with no water-mediating H-bond between Ser27@OG and UQ@O3. There was at least one water-mediating hydrogen bond appeared between them during the simulation. Water molecules that went into the binding site create a polar environment in the binding site which agreed well to the condition for electron transfer process in the Krebs cycle. However, we were not able to find any static water molecule which might be responsible for the interaction between UQ and Ser27@OG. All the waters appeared around the binding pocket and the longest occupancies were not more than 2 ps. This corresponded well with the RDF analysis.

**Figure 14:**
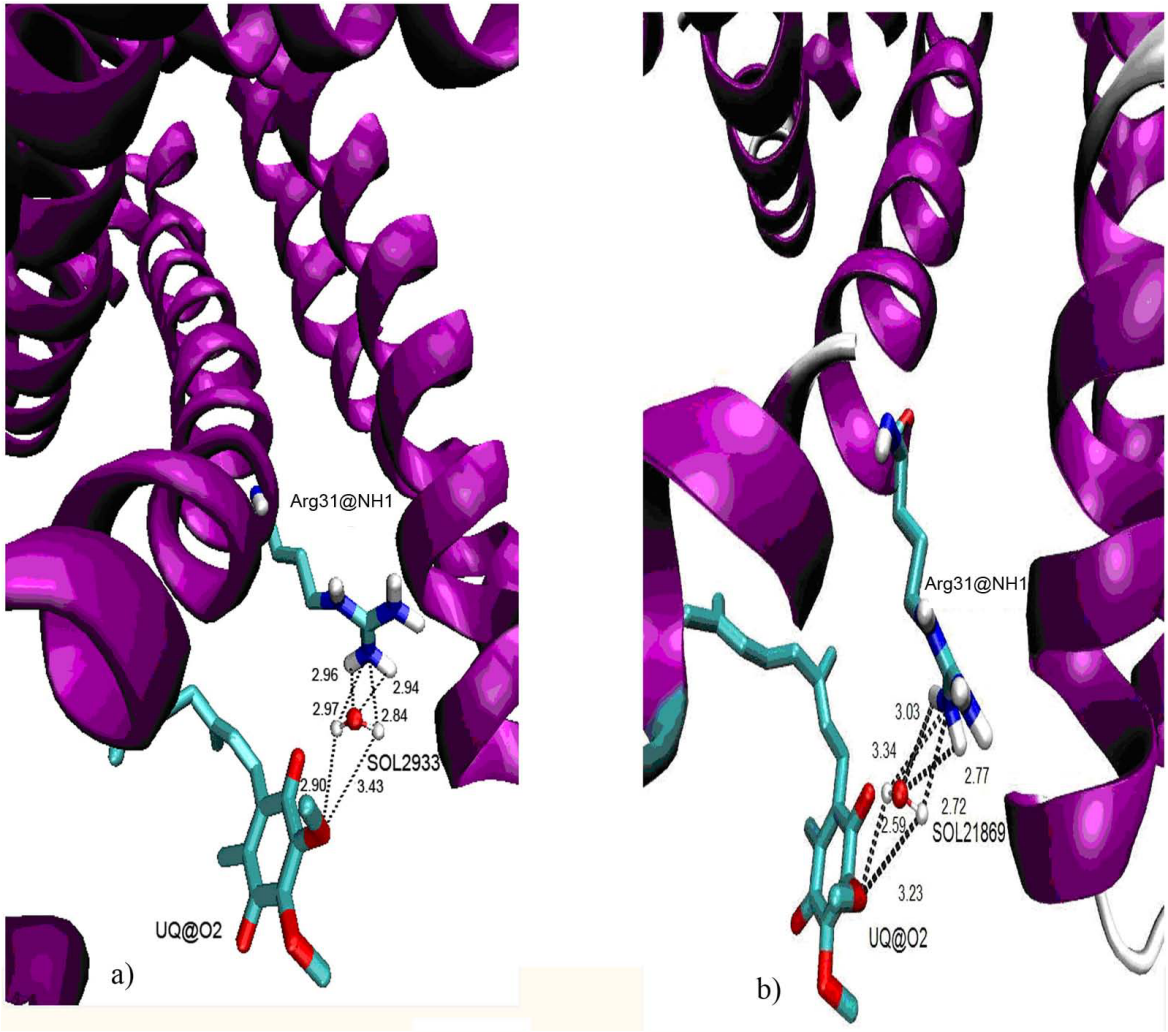
**Water molecules that found contributed to water mediated hydrogen bond**. (a) and (b) Two particular water molecules, namely SOL2933 and SOL21869, were observed to mediate hydrogen bond formation between UQ and protein during the production simulation time of 6.5 ns to 8.6 ns and 9 to 10 ns.

83.2% of the trajectory had at least one water-mediating H-bond at Arg31. However between UQ@O2, 68.8% of the trajectories were not able to form H-bond with water. Only about one third of the trajectory consisted of not more than 2 water-mediated H-bonds. The possibility of both interacting atoms to have a water-mediating H-bond was much lower as compared to Ser with UQ. This result did not correlate with the RDF result. However, two molecules of water were found sandwiched between Arg31@NH1 and UQ@O2 (Figure 14a and b).

**Table 5:** Hydrogen bond analysis between those interacting residues and UQ with water within 5Å of the interacting atom

**No. of hydrogen bond (HB)**	**5Å around SER27@OG**		**5Å around UQ@O3**		**5Å around ARG31@NH1**		**5Å around UQ@O2**	
	
	**No. of trajectory**	**(%)**	**No of trajectory**	**(%)**	**No. of trajectory**	**(%)**	**No. of trajectory**	**(%)**
	
0-HB	133	0.74	122	0.68	3025	16.81	12388	68.82
1-HB	2673	14.85	4881	27.12	4092	22.73	5333	29.63
2-HB	7090	39.40	10065	55.93	4632	25.74	270	1.50
3-HB	6048	33.61	2682	14.90	3627	20.15	9	0.05
4-HB	1611	8.95	240	1.33	1871	10.39	0	0
5-HB	357	1.98	10	0.05	570	3.17	0	0
6-HB	78	0.43	0	0	153	0.85	0	0
7-HB	10	0.05	0	0	24	0.13	0	0
8-HB	0	0	0	0	4	0.02	0	0
Total	18000	100	18000	100	18000	100	18000	0

### Functional implication derived from MD simulations

Oxidation of succinate to fumarate and reduction of UQ in the mitochondrial respiratory chain are carried out by SDH. Both processes utilized protons, H^+^. Due to SDH electroneutrality, it does not generate a proton motive force during catalysis. However, it formed a complex electron relay system which generate chemical energy through create proton gradient environment across the TM. Thus, the polar environment at the UQ binding site is very important in creating such a proton motive force during the catalysis of SDH [23]. Water molecules and the polarity of the interacting amino acids residues might have contributed in creating a polar environment. In the crystal structure of the template, Asp95 and Glu101 at chain C of SDH, Gln78 from chain D of SDH are located at the fringe of the water channel. These residues operate as a proton wire connecting the cytoplasm to the UQ binding site. Mutations studies were done in all these residues in order to eliminate the potential H-bond formation in water channel and altered the H-bonding network by manipulating the side chain [23]. Substitution of Asp95 to Glu95 on chain C extended the side chain which might lead to the interruption of H-bonding network in the proposed water channel. Substitution of Glu101 to Leu101 (chain C) and Gln78 to Leu78 (chain D) had created a hydrophobic environment which inhibited the formation of H-bonds. The reduction of SDH turnover rate was observed in all these generated SDH variants [23]. It has been demonstrated that in a pH8 environment where the H^+^ concentration in the cytoplasm decreased 90%, the enzyme turnover rate had decreased markedly [23]. However, all these mutations did not suppress the growth of the *K. pneumoniae* entirely. Hence, they proposed the existence of an alternative proton wire or pathway which involves the Asp15 residue from chain D of SDH [23].

**Figure 15:**
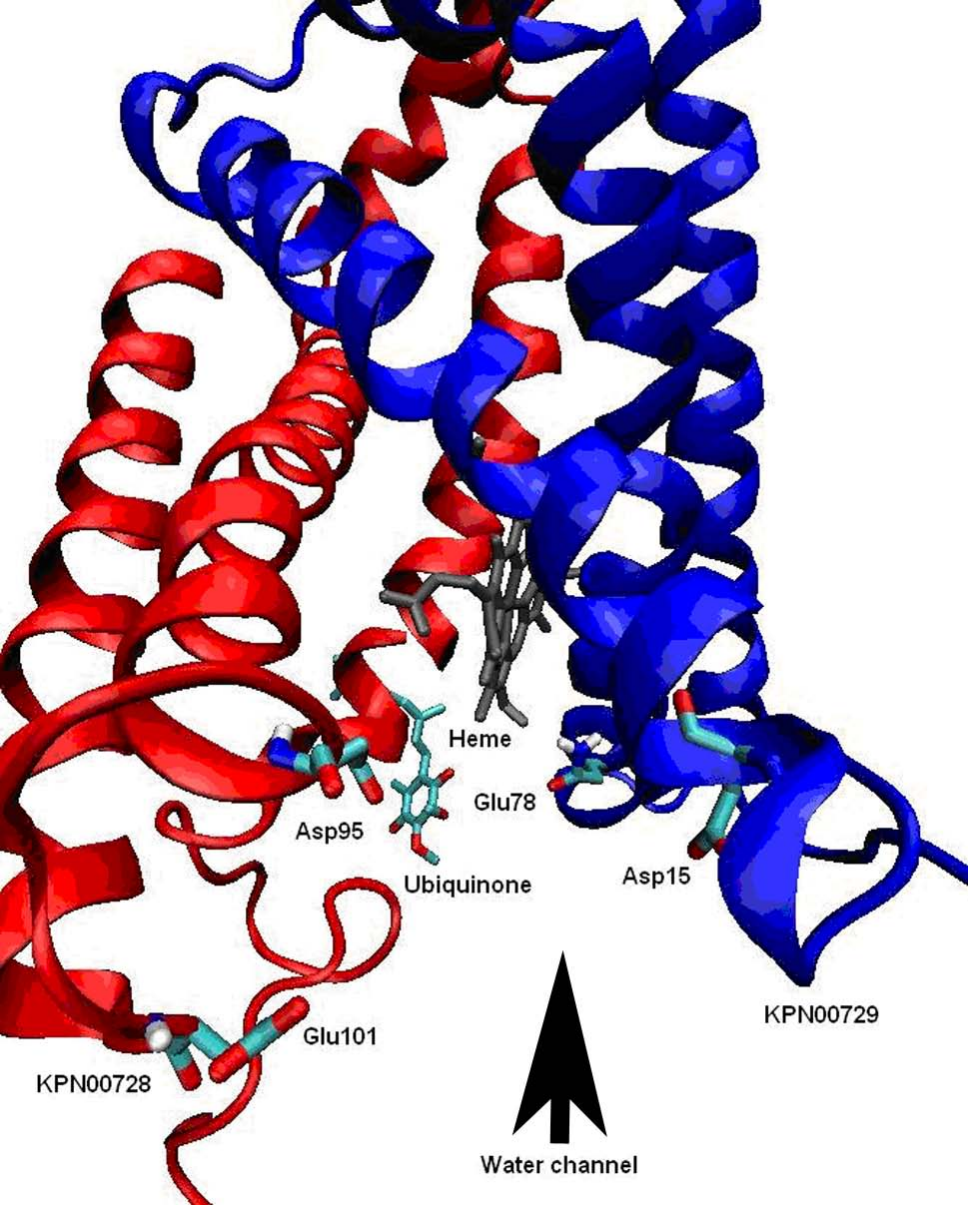
**A postulated water channel between KPN00728 and chain D of SDH.** Postulated water channel is indicated with black arrow. Residues such as Glu101, Asp95, Glu78 and Asp15 were found conserved. These residues had created a polar environment that might be able to aid the proton transfer process.

In our studies, Asp95 and Glu101 from KPN00728 and Gln78 and Asp15 from chain D of SDH were found to be conserved and located at the periphery of the membrane head group which connects with the cytoplasm (Figure 15). All of these residues have ionizable side chains and hydrophilic characteristic. This polar environment can contribute to the creation of the proton gradient across the mitochondrial membrane and UQ binding site connection. In addition, the heme group which is embedded deeper in the lipid bilayer needs a polar environment for its electron. Hence, polar residues like Asp and Glu from both chains are postulated to serve as proton transfer wires responsible in transferring protons from the cytoplasm to across the membrane.

UQ has two carbonyl and methoxy groups and one hydrophobic carbon tail. In our docking simulation, the positions of the carbonyl and methoxy groups of UQ were facing toward the protein. Strong H-bond was observed between carbonyl O4 and Tyr83 from chain D. On the other hand, the O1 carbonyl from UQ drifted toward the entrance of the water channel and was surrounded by water molecules. This solvation effect was most probably caused by 2 electron lone pairs of the carbonyl group. As for the hydrophobic carbon tail of UQ, it remained at the inner side of the entrance by avoiding the water molecules as shown in Figure 12. No significant changes in the orientation of the carbon tail were observed.

## Conclusions

In our present study, MD simulation was used to give further insight into the functionality of our built model of KPN00728 hypothetical protein from *Klebsiella pneumoniae* MGH78578 as chain D of SDH. This was achieved by investigating the dynamics of its interaction with UQ and chain D of SDH across a transmembrane environment which was successfully established in this study. The stability of the simulation correlated well with major experimental parameters which are important for dynamic study of binding interaction of UQ and SDH. Both Ser27 and Arg31 had failed to demonstrate the possibility of forming H-bond with UQ. However, interestingly, analysis on simulation trajectories indicated that water-mediating H-bond did indeed exist and was found sandwiched between Arg31@NH1 and UQ@O2. Water molecules also appeared to be around Ser27. The occurrence of these water molecules around the binding site of UQ indicated that they might be responsible for the interaction involving binding of UQ to SDH. Examination of the structural properties at the binding site revealed that polar residues such as Asp95 and Glu101 (KPN00728), Asp15 and Glu78 (chain D SDH) were conserved and located at the entrance of the channel believed to be a water channel. The polarity of these residues might create a proton motive force which is responsible in transferring protons from the water channel or cytoplasm. The observation of this MD study had provided conclusive evidence that KPN00728 is indeed part of SDH.

## Methods

### Setup of the simulation system

**Figure 16:**
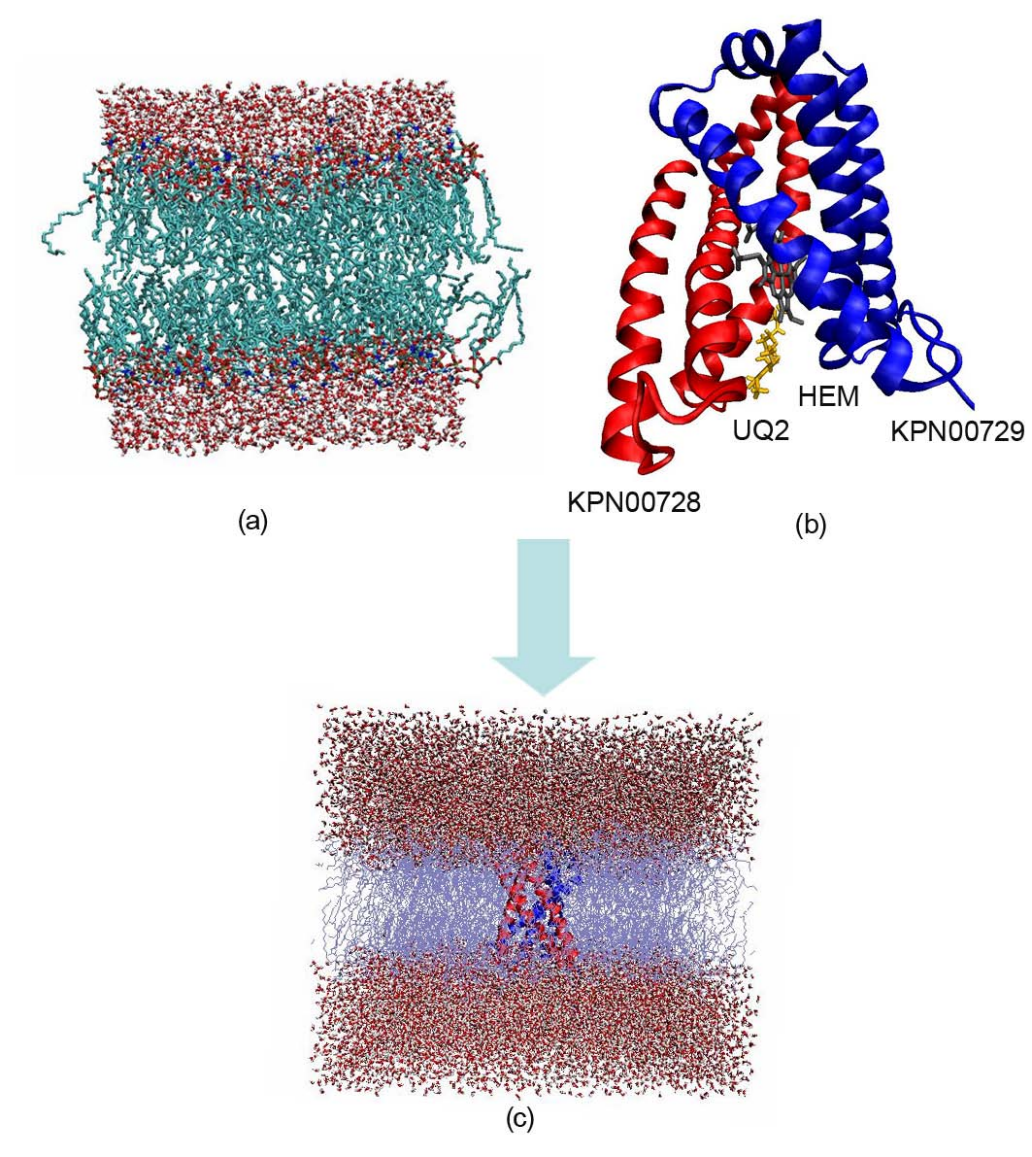
**Illustration on insertion of built model in pre-equilibrated POPC.** (a) 128 POPC, 2460 waters from Peter Tielemen. (b) Built model with docked result as a starting structure. (c) Built model was inserted into a duplicated block of 128 POPC.

A membrane bilayer simulation system which consisted of 512 palmitoyl oleoyl phosphatidyl choline (POPC) generated from 128 pre-equilibrated POPC obtained from Peter Tielemen’s website [10] was used to encapsulate the model built previously using genbox command from GROMACS 4.0.5 [24] (Figure 16). More than 90 molecules of POPC were removed from the constructed bilayer in order to accommodate the proteins. Topology file of UQ and heme were created using PRODRG2 server [25]. Gasteiger approach [26] was used to calculate partial atomic charges in UQ to ensure that the system is consistent with the molecular docking simulation carried out previously [5].

A total of 29153 single point charge (SPC) waters were added into the simulation box with the thickness of ~27 Å away from the lipid headgroup. Three counterions Cl^-^ were added to compensate for the net charge of the system, resulting in the system to be comprised of 111826 atoms. A total of 32,632 minimization steps were performed, starting with steepest descent (SD) and ended with conjugate gradient (CG), to remove unfavourable contacts. Subsequently, the system was subjected to equilibration in two phases. NVT equilibration was done to equilibrate the temperature of the entire system using Berendsen temperature coupling for 200 ps [27], whereby the protein complex was under position restraint condition. Then, NPT equilibration of the system on the protein complex was done for 2 ns under restraint condition. Nose-Hoover thermostat [28] was used to produce a correct kinetic ensemble and to allow molecular fluctuations within the system for more natural dynamics simulation. Semi-isotropic pressure coupling was applied. Upon completion of the two equilibration phases, the system was well equilibrated at the desired temperature and pressure. This was followed by the production run without any restraint. A total of 18 ns production MD was performed using an NPT ensemble.

### Simulation protocols

Molecular dynamics simulation of the built model/membrane protein was performed using GROMACS v4.0.5 package [24] under NPT ensemble at a pressure of 1 bar and temperature of 300K. The GROMOS96 53a force field was used for both built model and lipid bilayer system [29]. All bond lengths were constrained to their equilibrium value using SETTLE algorithm for water [30] and LINCS algorithm for the bonds between heavy atom and hydrogen atoms in protein, lipids and peptides [31]. Integration time step of 2 fs was used and the neighbour list to calculate non-bonded interaction was updated every 10 time steps during the entire simulation time. A cut-off of 12 Å for Coulombic and van der Waals interactions was applied. Correction of long range electrostatics was done using Particle Mesh Ewald method (PME) [32] with a fourth-order spline interpolation and Fourier grid spacing of 0.12 nm. Periodic boundary condition in all directions was applied in the simulation.

During NVT equilibration simulation, Berendsen temperature coupling method [27] was used with a temperature coupling constant (τT) of 0.1 ps. Each group (peptide, lipids, solvent/ions) was coupled to a separate temperature bath. Subsequently, in NPT equilibration and production simulation, pressures were applied independently using Parrinello-Rahman pressure coupling approach [33,34] in the x and y directions. To pack the lipids around the peptide and accelerate equilibration, a weak pressure coupling of 1.0 bar is given in the x and y directions with a pressure coupling constant (*τ*_P_) of 5.0 ps.

## Competing interests

The authors declare that they have no competing interests.

## Authors' contributions

**SBC:** simulations, analysis of simulation data, drafting, drafting figures and editing of the manuscript. **YMN:** discussion and editing of the manuscript. **HAW:** coordinated the study, discussion and editing the manuscript.
